# Results of Femtosecond Laser-Assisted Descemet Stripping Automated Endothelial Keratoplasty

**DOI:** 10.1155/2017/8984367

**Published:** 2017-06-11

**Authors:** Mohamed H. Hosny, Ayah Marrie, M. Karim Sidky, Sherif GamalEldin, Mohsen Salem

**Affiliations:** Department of Ophthalmology, Cairo University, Cairo, Egypt

## Abstract

**Purpose:**

To evaluate femtosecond laser in DSAEK surgery as an improvement to manual DSAEK.

**Settings:**

Department of Ophthalmology, Cairo University.

**Design:**

A retrospective observational clinical study.

**Methods:**

20 eyes with SBK and Fuchs' dystrophy underwent a Femto-assisted DSAEK by laser cutting of two matching posterior stromal discs in the recipient and donor corneas and then fitting the donor disc in the posterior corneal defect of the recipient using Busin's glide or Terry forceps.

**Results:**

Corneal thickness decreased significantly from a mean of 900-micron preoperative values (900.7 m) to 562 m postoperatively. Evidence of side healing was documented by OCT. One patient had a double AC, one patient had an air interface entrapment “Double Bubble,” one patient had a fungal infection and was treated by a therapeutic penetrating keratoplasty, and one patient had a CMO.

**Conclusion:**

Femtolaser-assisted DSAEK may be superior to manual techniques as it offers better centration, thinner graft/host complex, earlier corneal detergecense, and stronger healing. This study was registered at Researchregistry.com with a UID: researchregistry2274.

## 1. Introduction

The endothelium is a single layer of cells present at the back of the cornea. Cell density at birth can be as high as 7500 cells/mm^2^, decreasing to an average of about 2500–2700 cells/mm^2^ in older adults. Endothelial cells are not capable of significant mitotic activity. The normal rate of endothelial loss after age 20 years is approximately 0.5% per year. Surgical trauma as pseudophakic and aphakic bullous keratopathy, inflammation, and corneal dystrophies as Fuchs' dystrophy can accelerate this normal aging loss. When the cell density reaches a critically low level of about 300–500 cells/mm^2^, fluid begins to accumulate within the cornea. As a result, the cornea loses its transparency and the individual suffers a reduction in vision [1].

Fuchs' endothelial dystrophy (FED) is a condition in which there is premature degeneration of corneal endothelial cells [2]. Descemet stripped automated endothelial keratoplasty (DSAEK) has become the preferred method of treating endothelial dysfunction, after penetrating keratoplasty (PKP) had long been the gold standard for treatment due to its limitations including delayed visual recovery, unpredictable refractive changes, ocular surface complications, and the risk of losing the eye to suprachoroidal hemorrhage. DSAEK provides faster visual recovery with less induced surgical astigmatism and with lower rate of intraoperative and postoperative complications [3].

The femtosecond laser technique allows completely new trephination procedures in penetrating and lamellar keratoplasty. Thus, it is easier to get a watertight wound closure intraoperatively, and due to the larger wound surface, wound healing is faster. In lamellar keratoplasty, the femtosecond laser enables the surgeon to cut to any depth in the corneas resulting in thin corneal donor buttons, for example, for DSAEK [4].

One of the main causes of the poorer than expected vision after microkeratome-assisted DSAEK was usually associated with the presence of folds or wrinkles that can develop in the graft as it conforms to the host cornea [5]. The eye banks do not measure the curvature of the donor cornea, and no attempt is made to match donor and recipient curvatures, so in some cases, the curvature mismatch may be substantial leading to wrinkles in the graft [6].

### 1.1. Why Femtosecond Laser in DSAEK

By removing a properly centered posterior disc of the recipient stroma and placing an identical disc of the donor cornea in its place, three challenges of classic DSAEK are overcame, namely, centration, where the newly placed disc is placed in an exact central location, and cannot move, and over thickness of the graft/host complex putting extra burden on the newly implanted endothelium to deterge the thick complex in classic DSAEK in contrary to FS-assisted DSAEK where the posterior-removed disc is replaced by the new tissue thus decreasing the final corneal thickness and facilitating detergence of the edema. Finally, the side cut healing is not present in classic DSAEK which provides proposed stronger healing and reduces the risk of graft detachment.

This study aimed to assess the early and one year outcomes of this novel technique by reporting the structural and functional effects of totally femtosecond-assisted DSAEK on bullous keratopathy and Fuchs' dystrophy.

## 2. Materials and Methods

This is a retrospective observational study applied on twenty eyes of nineteen patients who underwent a total femtosecond-assisted DSAEK. This study was carried out from November 2015 to January 2016. Inclusion criteria were eyes with pseudophakic corneal decompensation <12-month duration and eyes with Fuchs' endothelial dystrophy.

Exclusion criteria were corneal dystrophies other than Fuchs', central/paracentral corneal scars, eyes with uveitis, glaucoma, or retinal vascular occlusive diseases, eyes with optic nerve diseases, and eyes with retinal detachments. The approval of Cairo University Ethical Board committee was obtained for the medical ethics and compliance with the Declaration of Helsinki for medical research. All patients were handed an informed consent to study and approve.

All patients underwent complete ophthalmological examination before surgery, including best corrected visual acuity (according to the Snellen VA chart), slit lamp examination, assessment of the IOP, endothelial cell density evaluation with specular microscopy, and corneal thickness with the anterior segment OCT and corneal pachymetry.

### 2.1. Surgical Technique

#### 2.1.1. Donor Tissue Preparation

Corneoscleral buttons of endothelial cell count not less than 2300 cells/mm^2^ were mounted on a disposable artificial anterior chamber. Infusion of balanced saline solution (BSS) to make the pressure high (between 60 and 65 mmHg) confirmed by applanating the anterior surface of the cornea, and online pachymetry is made to measure the central conreal thickness (CCT). A 200 KHz femtosecond laser (Alcon Wavelight FS200 Femtosecond Laser (Alcon Surgical, Fort Worth, TX, USA)) was used to resect the posterior stromal tissue. The laser is programmed to make a 150 *μ*m thickness lenticule from the endothelial side measured from the central cornea, of a diameter of 7.50 mm and an angle of 90°.

#### 2.1.2. Recipient Tissue Preparation

The patients' corneal thickness was measured preoperatively by anterior segment OCT (DRI OCT Triton, Swept source OCT; Topcon Corporation, Tokyo, Japan) and Scheimpflug tomography (Wavelight Allegro Oculyzer, Alcon Laboratories Inc., Erlangen, Germany), and after application of topical anesthesia and removal of the epithelium, the CCT is remeasured by online pachymetry using the Wavelight EX500 Excimer Laser (Alcon Laboratories, Inc., Fort Worth, TX, USA). In the first 13 patients, the removed posterior corneal discs were dissected by the femtosecond laser exactly as the donor's cornea by the same technique and depth. In the last 7 patients, the removed posterior corneal disc was aimed to be 120 microns and the reason will be explained later.

Then, the patient was sent to the operative theater where they are given a peribulbar anesthesia; sterilization of the skin by povidone-iodine (betadine) 10% draping of the eyelids and the eyelashes and then conjuctival wash with betadine 5% is made. First, a 20-gauge MVR incision is made at 6 o'clock through which a trypan blue 0.06% is injected to delineate the precut lenticule. Then, an anterior chamber maintainer has been inserted through this incision attached to a bottle of BSS with a bottle height that gives a 20 mmHg pressure. Then, a 2.80 mm keratome incision is made at 12 o'clock, anterior chamber wash by BSS with the anterior chamber maintainer is turned on, an inverted (reversed) Sinskey hook is brought to the anterior chamber to dissect the remaining attachments of the precut lenticule, and the lenticule is withdrawn by a toothed forceps from the anterior chamber. The extracted lenticule is then inspected in front of the patient's cornea to make sure that there are no missing parts. The donor's cornea is then inverted so that the endothelial side becomes up; then by the microforceps (End-gripping forceps), the precut lenticule is stripped from the donor's cornea and the endothelial side is covered by dispersive OVD.

In 10 cases, the lenticule was inserted by a Busin's glide, and in the other 10 cases, the lenticule was inserted by a taco fold using Terry forceps as the procedures were performed by 2 surgeons each with a preferred surgical technique.

#### 2.1.3. Busin's Glide Technique

A 5 mm keratome incision is made at the nasal part of the patient's cornea, and a MVR incision is made just opposite to it. The Busin's glide has then been put just at the 5 mm keratome incision while the forceps cross the anterior chamber from the MVR incision and get out of the keratome incision to grasp the lenticule at that stage when the anterior chamber maintainer is turned off. The forceps withdraw the lenticule to the anterior chamber, and as soon as the lenticule is in the anterior chamber, the irrigation is turned on so the jet of BSS helps in unfolding of the lenticule. The fluid flow will push the lenticule to the back of the patient's cornea, and then the keratome incision is closed by 10-0 sutures.

#### 2.1.4. Terry Forceps Technique

The lenticule is folded 40%/60 and held by the forceps; it is then introduced from a superiorly placed 5 mm keratome incision; and as the forceps is opened and withdrawn, the irrigation is turned on to facilitate the unfolding of the lenticule. The incision is then secured with 10-0 sutures.

A big air bubble is then injected in the anterior chamber, and milking of the lenticule from above the cornea is then made to move the lenticule until it fits exactly in the posterior hole. After 15 minutes, the air bubble is reduced so papillary block does not occur. The patient remains strictly face up for 24 hours. The patient is then examined on the slit lamp next day where the lenticule position and the presence or absence of a double anterior chamber are checked. Follow-up was weekly for one month and then every month for 6 months and at 9 months and 12 months.

In all follow-up visits, the patients underwent slit lamp examinations, IOP measurement, and anterior segment OCT.

#### 2.1.5. Statistical Analysis

The data were statistically described in terms of mean ± SD, median, correlation, and percentages when appropriate comparison of numerical variables between the two study groups was done using Wilcoxon signed-rank test while correlation between many groups was performed with Pearson correlation. *P* values < 0.05 were considered significant. All statistical calculations were done using computer programs IBM® SPSS® Statistics 21 (Statistical Package for the Social Science) (SPSS Inc., Chicago, IL, USA).

#### 2.1.6. Results

This was an interventional prospective case series study applied on twenty eyes of nineteen patients who underwent a total femtosecond-assisted DSAEK between Nov 2014 and Dec 2015. Sixteen eyes were pseudophakic at the time of DSAEK and two aphakics. In two patients, DSAEK was combined with phacoemulsification. One patient had a phakic anterior chamber IOL that causes the corneal decompensation that was removed, phacoemulsification was done, and IOL was implanted in the bag. One patient with Fuchs' dystrophy had DSAEK combined with phacoemulsification. The median age of patients was 61, 11 were females and 8 were males.

The mean of the endothelial cell count of the donor's corneas used was 2500 cells/mm^2^. VA has been measured in Snellen, and they were converted to a logarithm of minimum angle of resolution (logMAR) to facilitate statistical analysis.

**Figure 1:**
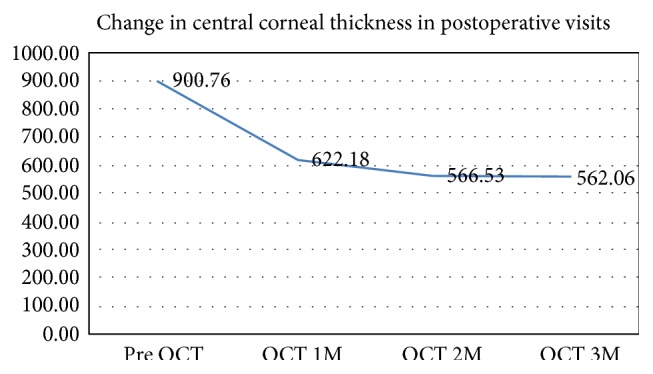
Change in central corneal thickness overtime from preoperative values to 3 months postoperatively.

Results of this study showed a significant improvement in the corneal thickness measured by anterior segment OCT (DRI OCT Triton, Swept source OCT; Topcon Corporation, Tokyo, Japan), with maximum decrease in the thickness in the first one month and to a lesser extent two and three months after the procedure (Figure 1, Table 1).

**Figure 2:**
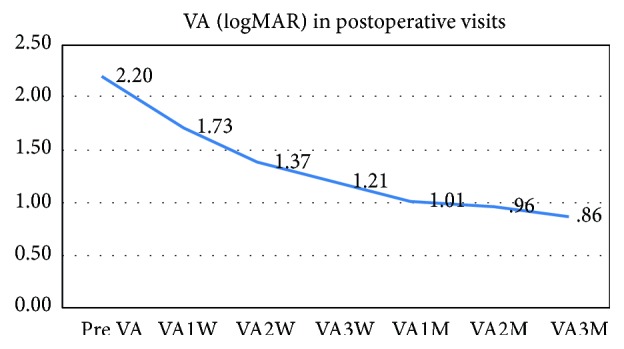
The change in VA (logMAR) after 1 week, 2 weeks, 3 weeks, 1 month, 2 months, and 3 months postoperatively.

Regarding the visual acuity, there was statistically significant improvement in visual acuity that was more significant in the first month postoperatively to a lesser improvement after two and three months with good correlation to the decrease in the corneal thickness (Table 2, Figure 2).

Table 3 showed statistical significance (*p* < 0.005) in visual acuity improvement when comparing the vision in each visit as compared to the preoperative vision, and when comparing each vision at each visit to the previous visit, except when comparing vision 2 months postoperatively to the vision one month postoperatively, there is improvement but not statistically significant.

**Figure 3:**
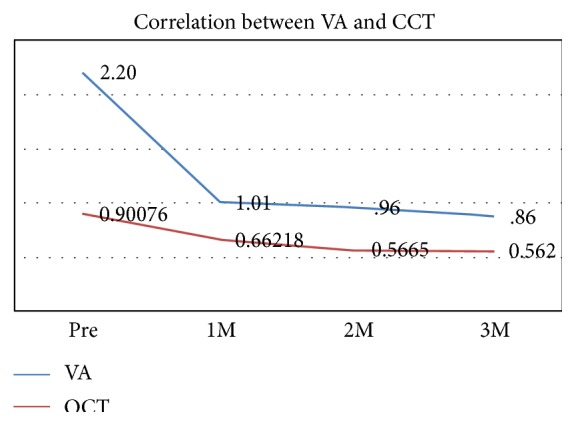
Correlation between change in VA (logMAR) and change in CCT after 1 month, 2 months, and 3 months after surgery.

There was no statistical correlation between the decrease in the corneal thickness and the visual improvement (Figure 3).

**Figure 4:**
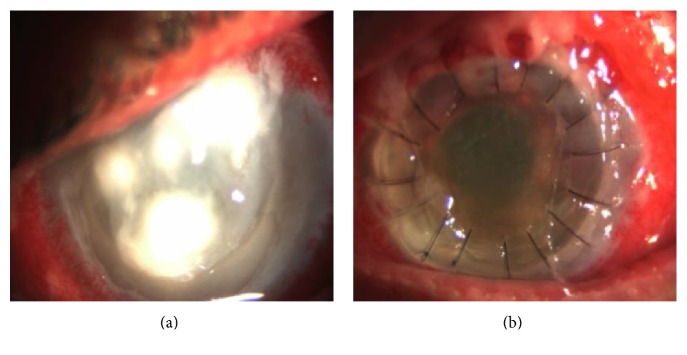
(a) Fungal keratitis post DSAEK. (b) Same patient after therapeutic penetrating keratoplasty.

One patient (*n* = 1, 5%) had postoperative fungal keratitis with corneal melting and had a therapeutic PKP (Figures 4(a) and 4(b)).

**Figure 5:**
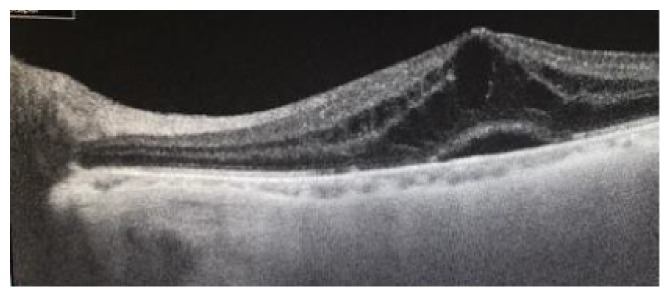
Cystoid macular edema with RPE detachment in one patient postoperatively.

One patient (*n* = 1, 5%) had cystoid macular edema with retinal pigment epithelium detachment that took place after two months of surgery and caused diminution of vision (Figure 5).

**Figure 6:**
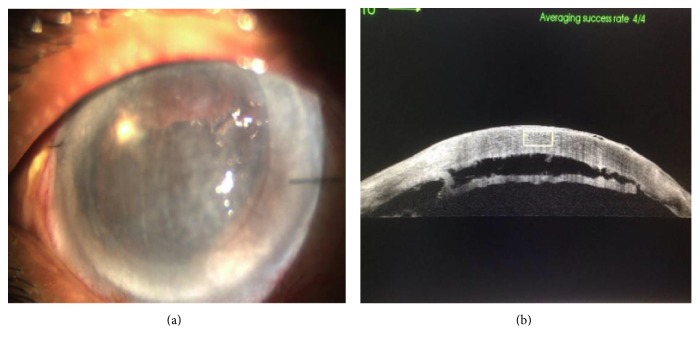
(a) Double anterior chamber in one patient. (b) OCT of the same patient.

One patient (*n* = 1, 5%) had a double anterior chamber discovered one day after surgery and confirmed by anterior segment OCT (Figures 6(a) and 6(b)).

**Figure 7:**
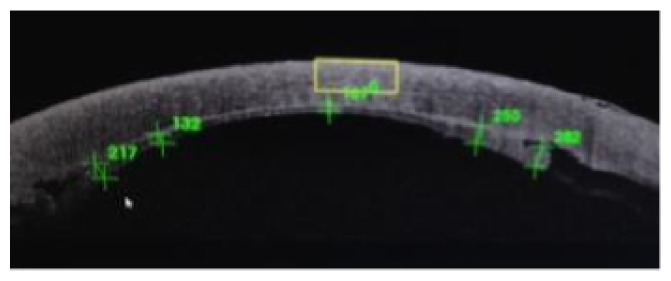
Incomplete reattachment after rebubbling.

After air reinjection, a small gap was still there (Figure 7).

**Figure 8:**
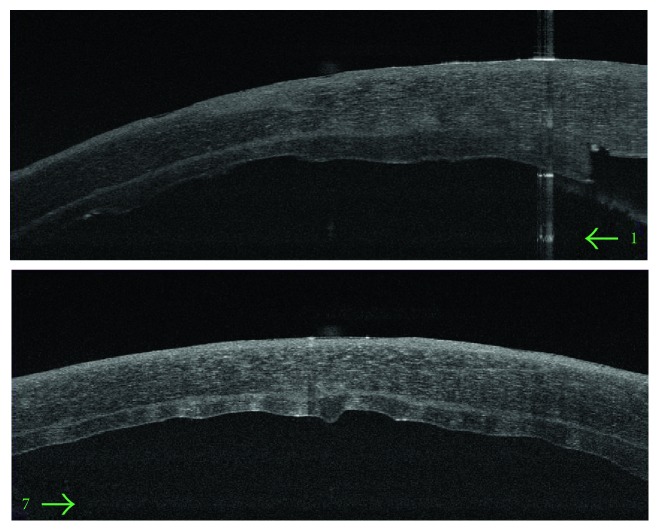
Complete reattachment after rebubbling for the second time.

So air injection for the third time took place, the lenticle was excellently in place, and then the lenticule was excellently in place (Figure 8).

#### 2.1.7. Complications Specific to This Prescribed FS-Assisted DSAEK Technique

We are describing two complications specific to this type of surgery, namely, the thickness disparity and the interface air trapping or what we termed the “Double Bubble.”

#### 2.1.8. Thickness Disparity

**Figure 9:**
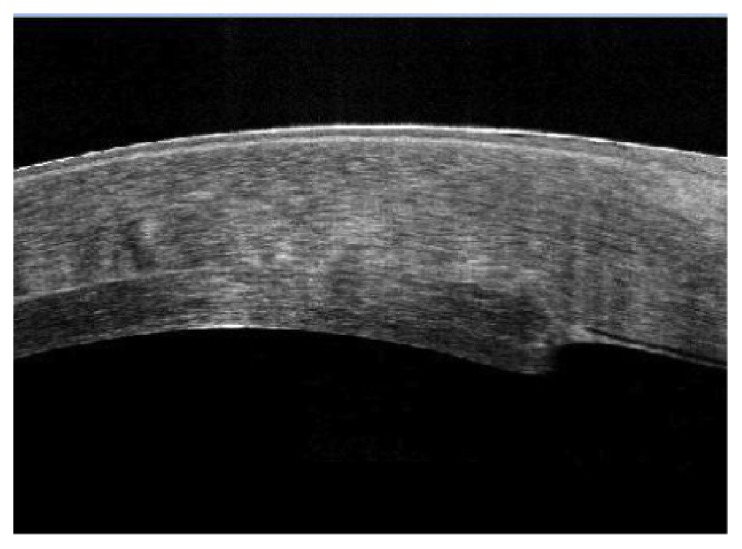
Severe disparity between the posterior graft and the posterior corneal defect one month postoperatively.

**Figure 10:**
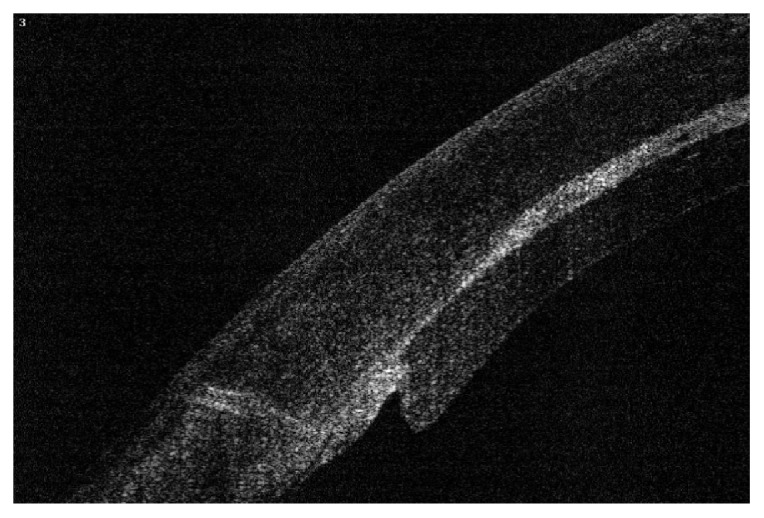
Another case with severe disparity between the posterior graft and the posterior corneal defect one month postoperatively.

**Figure 11:**
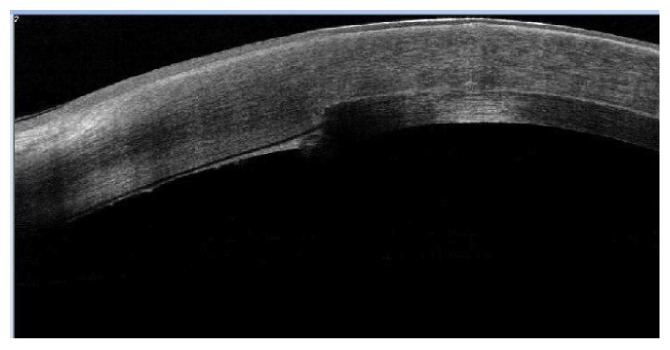
Much better match one month postoperatively after parameter modification.

**Figure 12:**
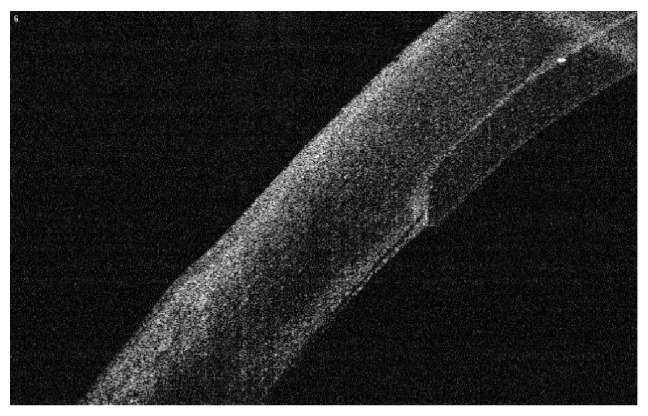
Another good match one month postoperatively after our parameter modifications.

In the first 6 cases, as we implanted 120-micron thick grafts equivalent to 120-micron defects, our postoperative observation over the first few weeks was the occurrence of thinning of the recipient cornea, and due to a lesser amount of edema in the implanted graft than the recipient cornea, the posterior defect cut in the recipient cornea became progressively shallower and the posterior disc protruded. This did not cause any change in the visual rehabilitation course but was evident by OCT. After the first six cases, we modified our parameters by cutting a 180-micron posterior defect and fitting it with a 120-micron graft, as the host cornea shrinks with time, both graft and its intended place seemed to match much better by OCT (Figures 9, 10, 11, and 12).

#### 2.1.9. Air Trapping in the Interface or the “Double Bubble”

**Figure 13:**
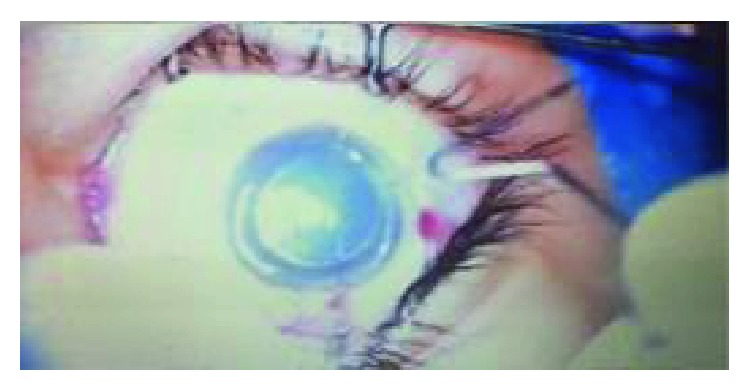
Air entrapment in the interface or “Double Bubble.”

**Figure 14:**
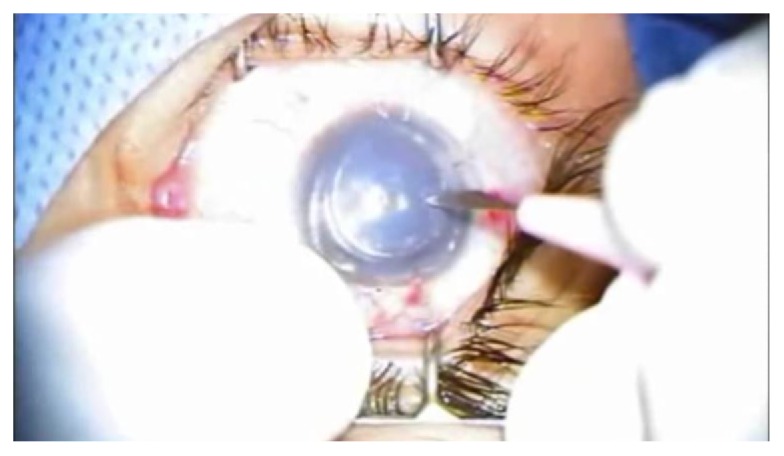
Venting performed to allow air to escape.

**Figure 15:**
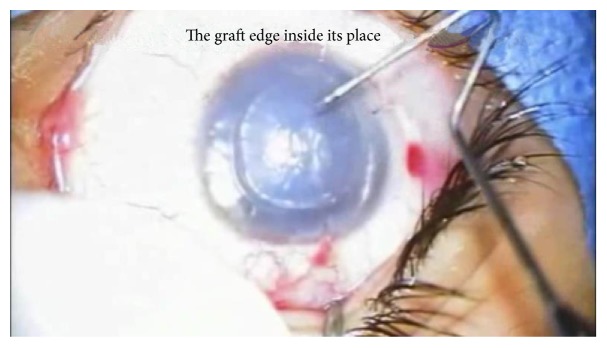
Reappearnce of corrugations denoting good apposition of the graft to the back of the stroma.

Again, this is a complication that is specific to this technique and cannot happen in manual DSAEK, as the posterior graft is placed in its place and after the anterior chamber is inflated with air, air can be trapped in the interface and stays there for up to 48 hours. This delays the early clearing of the corneal edema and should be suspected if there is significant persistent edema on the second day postoperatively and can be confirmed with OCT. This is usually suspected at the end of the surgery if after air injection there is absence of the normal corrugations seen on the back side of the cornea denoting the presence of a “Double Bubble.” If discovered at the end of the surgery or on the second day, venting should be carried out to allow the air to escape and adhere the graft to the host cornea in order for the implanted endothelium to work. This complication happened in two cases in this series: one was discovered at the end of the surgery and the other on the second day. Both underwent venting with immediate successful attachment of the graft (Figures 13, 14, and 15).

#### 2.1.10. Evidence of Side Cut Healing

**Figure 16:**
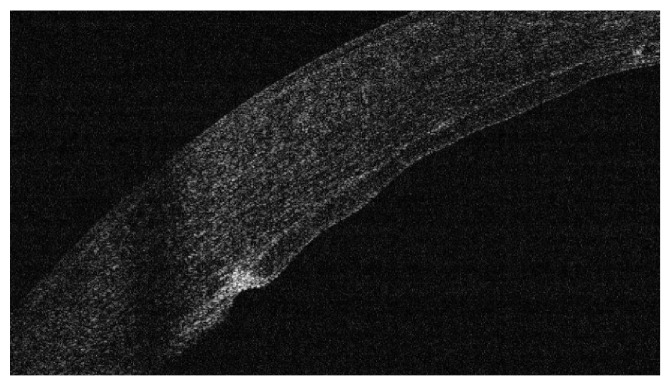
Evident side cut fibrosis by OCT one year after surgery.

**Figure 17:**
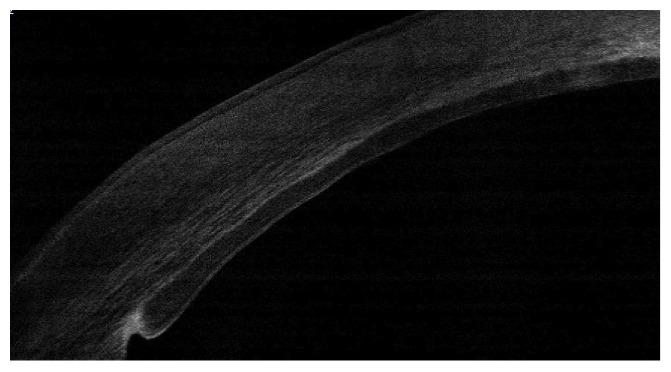
Another OCT showing side cut fibrosis and healing.

As an important point in the hypothesis of this technique is the side healing leading to better stability and stronger attachment of the implanted graft, we investigated the presence of side healing by OCT appraisal one year after the procedure. Evidence of side cut strong attachment was found in all cases in the form of side cut fibrosis (Figures 16 and 17).

### 2.2. Discussion

In Fuchs' dystrophy and bullous keratopathy, DSAEK became the standard treatment but with the most frequent complications being dislocation-detachment of the lenticule and, to a lesser extent, endothelial rejection [7].

In a report by the American Academy of Ophthalmology to evaluate the safety and outcome of DSAEK by Koenig and Covert in 2007 showed that the mean incidence of graft dislocation is 14%; range, 0%–82% [8]; the main outcome of our study is to address that complication and improve the results by cutting the recipient graft by the same depth and diameter of the donor's lenticule by the femtosecond laser so that the lenticule lodges to its place decreasing the incidence of graft dislocation and detachment and with proper centration.

In a study by Hjortdal et al. in 2012 which is to evaluate the femtosecond cutting for the donor's graft, they had to do rebubbling of air in 5 out of 10 patients to manage graft completely and partial detachment [9], but in our study, we had one graft detachment (5%) that needed rebubbling that was due to insufficient air tamponading in a vitrectomized globe and another patient with decentered graft (5%) that was due to incomplete methyl removal following cataract extraction that was made simultaneously with the DSAEK. Basak SK and Basak S in 2014 studied the complications of DSAEK and had graft dislocation in 21% of cases with failure of rebubbling in 25% of the dislocated cases [10]; we had better results in our study as we have no failure in rebubbling as the main advantage of our study is the prominent side healing as it may be speculated that the parallel organization of the collagen fibers in the posterior part of the stroma generate tiny collagen fibril strands when the tissue is cut during femtosecond laser-generated plasma formation [9].

In comparison with the DMEK, Ham et al. 2009 reported that 10 of 50 cases required a secondary DSEK procedure because of complete or partial DMEK detachment [11], but this complication rate decreased notably as surgeons gain experience, Dirisamer et al. [12].

In a multicentric trial including 5 centers in the Netherland, Cheng et al. found that stray light and contrast sensitivity improved over the postoperative months after FS-DSAEK and are comparable to PKP results. In our study, although we did not perform stray light testing or contrast sensitivity, we did not notice any interface problems over the follow-up period that would lead to a decrease in BCVA. However, the lesser improvement in BCVA might be due to subclinical changes in the interface due to irregularity in the femtosecond laser cut.

As for the currently used manual technique for the DSAEK, there is an increase in corneal thickness because posterior donor stroma is added without removal of any recipient stroma [13] in our study where an equal stroma is removed from the recipient's cornea not causing an extra burden with the overly swollen edematous cornea on the newly implanted endothelium.

Regarding DMEK which also does not increase the total corneal thickness but is challenged by the fact that Descemet's membrane is quite fragile and is implanted without attached stroma to provide support, a significant percentage of donor corneas is lost while harvesting the membrane or by subsequent primary graft failure. DMEK needs a well-experienced surgeon with a steep learning curve [14].

In a study by Mencucci et al. in 2015 who documented the histological finding of a corneal button removed from a patient after DLEK, they found a fibrotic repair limited to the peripheral margins that gives advantage for DLEK over DMEK in a form of postoperative lenticule stability [15].

The main problem with the side sealing of the graft/host junction is that air can be trapped between the graft and the posterior stroma, causing what we call a double bubble sign; this may lead to delay in corneal clarity over the first 48 hours and compromises graft to stroma attachment in the early postoperative period, and this problem is treated by venting, a positive sign for venting success is the corrugation at the donor's lenticule and that the donor's lenticule fits in its place properly.

Another issue we faced was the thickness disparity when cutting the posterior recipient defect with the exact thickness as the graft; you have a perfect match in the immediate postoperative period. But over time, the implanted graft succeeds in clearing the overlying stroma from its edema. So the cornea shrinks, and the posterior defect becomes shallow. This can lead to minimal graft protrusion over time, which is still better than the total graft add on in manual DSAEK. This is overcame by cutting a deeper posterior defect than the graft thickness (180 m for a 120 m graft).

There were no donor lenticule preparation complications that were reported in cases of manual dissection or microkeratome preparation like excessively thickened donor posterior lenticules and donor tissue perforation [16–18].

We had four recipient corneas with uneven femtosecond cut through their cornea and even areas with no cleavage where the descemet and endothelium were removed manually and that was due to the uneven corneal thickness with bullous keratopathy especially in long standing corneal edema; this may be due to the nonequal separation of the corneal lamella by the water pressure. This is compounded by any attempt of stromal fibrosis. This is evident also in areas with thickness above 1200 *μ*m, (as that is the upper limit for the femtosecond penetration). As we implant the perfectly regular graft in the defect, it can be deeply imbedded in one part and flush or slightly protruding in another, but that complication reduced after we added the use of the corneal thickness and femtosecond application after removal of the epithelium. The corneas with the uneven cut did not show lenticule detachment.

The recipient corneas were cut for thickness of 180 *μ*m to give a range for that disparity of the cornea as a thinner parameter may miss a part of the cornea, and as we get deep in the cornea, the laser becomes less effective as the laser energy gets more scattered [19].

The patients with lesser preoperative corneal thickness had a smoother and easier recipient's graft separation due to better penetration of the femtosecond laser; also, patients with a lesser period of pseudophakic bullous keratopathy showed a more uniform femtosecond laser cut and easier separation, as long-standing edema causes anterior stromal haze and stromal scaring, as shown by the confocal microscope [15].

Suh et al. in 2008 also studied the complications of DSAEK and had 5% cystoid macular edema developed [17]; in our study, we had also 5% development of cystoid macular edema that was developed 2 months after surgery and we had one patient with corneal abscess and melting that developed one week after surgery and with a penetrating keratoplasty; the pathology showed acute inflammation with fungal hyphae confined to the graft, and the patient's own cornea showed acute inflammation.

In our study, we had a correlation between the decrease in the corneal thickness and the visual acuity improvement but was not statistically significant.

Visual and refractive outcomes have made the EK the treatment of choice for endothelial dysfunction. Whereas PK typically causes a 3-4D increase in mean refractive cylinder, EK causes little to no change from the preoperative mean [20].

However, a transient increase in manifest cylinder may occur if sutures are used to close the incision. EK likewise causes either no change in mean spherical refraction or just a mild hyperopic shift. Spherical equivalent outcomes can be influenced by the donor dissection technique. A manual donor dissection on an artificial anterior chamber tends to produce a shallower depth in the periphery, resulting in a meniscus-shaped donor lenticule, which can cause a hyperopic shift [20].

Many microkeratomes tend to cut deeper in the periphery, and since the cornea is deeper in the periphery, this can result in a relatively planar central donor lenticule [21].

In our study, cutting the donor's and the recipient's lenticules (on an artificial anterior chamber) with the femtosecond laser after the docking takes place gives a slightly deeper peripheral lenticule thickness.

## 3. Conclusion

Femtolaser-assisted DSAEK may prove to be a better technique, in that, it provides better side stability and attachment, thinner graft/host complex, and hence faster clearance of preoperative edema and better graft centration. The only unique complication is air entrapment in the interface that can be managed by venting.

### 3.1. What Was Known

Consider the following:

- DSAEK is the most tested form of endothelial keratoplasty in corneal endothelial dysfunction.
- Postoperative graft detachment is an important postoperative complication.
- The thick graft/host complex can delay clearing of corneal edema.
- Decentration of the graft may lead to low visual results.

### 3.2. What This Paper Adds

Consider the following:

- Fitting the graft in a posterior matching defect will stabilize the graft.
- This will decrease the graft/host thickness and facilitate clearing of corneal edema.
- Side healing offered by this technique provides strong attachment to the host.
- This can be done easily by cutting both the donor and the posterior host corneas with femtosecond-assisted laser.

## Figures and Tables

**Table 1:** The mean, standard deviation, median, maximum, and minimum for the visual acuity and central corneal thickness (pre VA: preoperative VA; W: week; M: month; OCT: central corneal thickness measured by OCT; pre OCT: preoperative OCT).

	Mean	Standard deviation	Median	Maximum	Minimum
Pre VA	2.20	0.69	2.07	4.00	1.40
VA1W	1.73	0.44	1.50	2.50	1.17
VA2W	1.37	0.12	1.40	1.50	1.07
VA3W	1.21	0.17	1.17	1.50	0.90
VA1M	1.01	0.21	1.00	1.40	0.60
VA2M	0.96	0.20	1.00	1.30	0.50
VA3M	0.86	0.16	0.90	1.00	0.50
Pre OCT	900.76	113.41	901.00	1097.00	733.00
OCT1M	622.18	39.92	632.00	689.00	560.00
OCT2M	566.53	26.74	561.00	624.00	500.00
OCT3M	562.06	37.38	557.00	645.00	455.00

**Table 2:** Statistical significance (*p* < 0.005) in central corneal thickness decreases when comparing the CCT in each visit as compared to the preoperative vision, and when comparing each vision at each visit to the previous visit, except when comparing CCT 3 months postoperatively to the CCT 2 months postoperatively, there is improvement but not statistically significant.

	OCT1M—pre OCT	OCT2M—pre OCT	OCT3M—pre OCT	OCT2M—OCT1M	OCT3M—OCT2M
*Z*	−3.622^b^	−3.621^b^	−3.621^b^	−3.621^b^	−1.398^b^
Asymp. Sig. (2-tailed)	0.0001	0.0002	0.0001	0.000	0.162

b = 1 billion.

**Table 3:** Wilcoxon signed-rank test based on positive ranks.

	VA1W—pre VA	VA2W—pre VA	VA3W—pre VA	VA1M—pre VA	VA2M—pre VA	VA3M—pre VA
*Z*	−2.842^b^	−3.627^b^	−3.629^b^	−3.624^b^	−3.624^b^	−3.625^b^
Asymp. Sig. (2-tailed)	0.004	0.0001	0.0001	0.0004	0.0003	0.0002

		VA2W-VA1W	VA3W-VA2W	VA1M-VA3W	VA2M-VA1M	VA3M-VA2M

*Z*		−3.685^b^	−3.302^b^	−3.638^b^	−2.135^b^	−3.237^b^
Asymp. Sig. (2-tailed)		0.0007	0.001	0.0001	0.033	0.001

b = 1 billion.
